# A universal metric for ferroic energy materials

**DOI:** 10.1098/rsta.2015.0303

**Published:** 2016-08-13

**Authors:** Ekkes Brück, Hargen Yibole, Lian Zhang

**Affiliations:** 1 Fundamental Aspects of Materials and Energy, Department of Radiation Science and Technology, Faculty of Applied Sciences, Delft University of Technology, Mekelweg 15, 2629 JB Delft, The Netherlands; 2 BASF Nederland B.V., Strijkviertel 61, 3454 PK De Meern, The Netherlands

**Keywords:** magnetocaloric, Fe2P, coefficient of refrigerant performance, reversible cycle

## Abstract

After almost 20 years of intensive research on magnetocaloric effects near room temperature, magnetic refrigeration with first-order magnetocaloric materials has come close to real-life applications. Many materials have been discussed as potential candidates to be used in multicaloric devices. However, phase transitions in ferroic materials are often hysteretic and a metric is needed to estimate the detrimental effects of this hysteresis. We propose the coefficient of refrigerant performance, which compares the net work in a reversible cycle with the positive work on the refrigerant, as a universal metric for ferroic materials. Here, we concentrate on examples from magnetocaloric materials and only consider one barocaloric experiment. This is mainly due to lack of data on electrocaloric materials. It appears that adjusting the field-induced transitions and the hysteresis effects can minimize the losses in first-order materials.

This article is part of the themed issue ‘Taking the temperature of phase transitions in cool materials’.

## 1. Introduction

Domestic refrigeration and air-conditioning contribute to more than 20% of the electricity bill of a US household [1]. In (sub)tropical areas like Singapore, this even exceeds 50% [2]. The majority of cooling devices nowadays utilize the vapour refrigeration cycle, which works as follows. First, the gas is compressed in a compressor, the heat produced in the compression stage is released to the environment and the gas condenses to form a liquid. In a throttling stage, the pressure of the liquid is lowered and the fluid cools down, forming a mixture of liquid and gas. Evaporation from the cold fluid takes up the heat from the substance that needs to be cooled and the gas is fed back to the compressor.

This refrigeration cycle can be made energy-efficient when certain gases are used. However, these gases are extremely strong greenhouse gases. Currently, refrigerant gases are the fastest-growing source of greenhouse gas emissions. If left unchanged, it is expected that in 2050 refrigerant gases will represent 9–19% of global greenhouse gas emissions [3].

A similar, but more energy-efficient refrigeration cycle than described above can be achieved with magnetic materials that show a large magnetocaloric effect (MCE). These materials heat up when a magnetic field is applied. After this heat is transferred to the environment, they cool down on removing the magnetic field and can take up heat from the substance that needs to be cooled. The processes as described are highly reversible, and therefore very energy-efficient, which can lead to a lower utility bill. Additionally, these magnetic materials are solids that can be recycled and do not contribute to the atmospheric greenhouse effect. Thus, this solid-state technology has the potential to strongly reduce the environmental impact of the present cooling technology.

**Figure 1.**
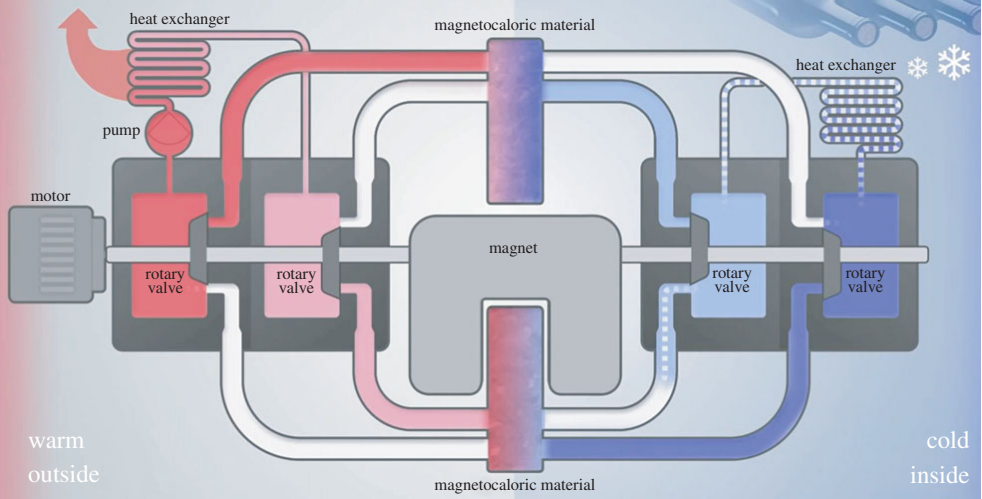
Schematic picture of a magnetocaloric wine cooler using MnFePSi type of materials.

Recently, a prototype of a full-grown appliance was presented at the Las Vegas Consumer Electronics Show CES2015 [4], as shown in figure 1. This appliance contains active magnetic regenerator (AMR) MCE materials developed at TU Delft and produced by BASF. It is the result of a collaboration between Haier, Astronautics Corp. and BASF. This prototype is an important step towards commercialization of this technology, but it also shows the complexity of such a machine. Obviously, expertise in quite different fields of technology is required to achieve good performance. One of the important steps is translating magnetic properties into thermodynamic performance. In this paper, we discuss a metric, the coefficient of refrigerant performance (CRP), to characterize MCE materials. CRP was originally introduced in 1985 by Wood & Potter [5] to MCE, but has hardly been applied to modern MCE materials.

## 2. Promising magnetocaloric materials

All magnetic materials show an MCE. This effect is usually enhanced in the vicinity of a magnetic phase transition. The relevant quantities for magnetic cooling are the field-induced temperature change Δ*T* and entropy change Δ*S*, respectively. The former represents the driving force for heat transfer and the latter represents the amount of heat that can be pumped in one refrigeration cycle. Obviously, both these quantities are a strong function of applied magnetic field and temperature.

For room-temperature applications, Gd metal with a Curie temperature of 293 K is considered as a benchmark material, as it has a rather large magnetic moment and displays a rather large Δ*T* in moderate fields [6]. Giant-magnetocaloric materials, such as FeRh [7], Gd_5_(Ge,Si)_4_ [8], Heusler alloys [9–11], La(Fe,Si)_13_ and its hydrides [12,13], MnAs [14], MnFe(P,X) compounds with X=As, Ge or Si [15–17] and to a lesser extent manganites [18], combine large Δ*T* and large Δ*S* values. However, as the phase transition in these materials is of first order, hysteresis effects can lead to a strong reduction of these values in a cyclic process. This becomes especially relevant if one wants to use these materials in a commercial cooling device, as the magnetic field that can be generated at reasonable costs is about 1 T [19], rather than the 5–14 T that are nowadays standard fields available in commercial magnetometers based on superconducting coils [20].

## 3. Current metrics

Another metric, the refrigerant capacity (RC), also introduced by Wood & Potter [5], is rather popular [21], in particular for second-order MCE materials [22]. From the fact that the second law of thermodynamics requires that the entropy change at the cold side Δ*S*_c_ cannot exceed the entropy change at the hot side Δ*S*_h_, they derive an expression for RC:
3.1
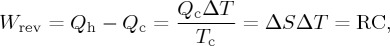
with *W*_rev_ the reversible work, *Q*_h_ and *Q*_c_ the expelled and received heat at warm temperature *T*_h_ and cold temperature *T*_*c*_, respectively, Δ*T*=*T*_h_−*T*_c_ and Δ*S*=Δ*S*_h_=Δ*S*_c_. In their paper, *T*_h_ and *T*_c_ are rather ill defined, and it has become a general practice to take Δ*T* as the width at half-maximum of a plot of Δ*S* as a function of the temperature [22]. Obviously, this metric is not dimensionless and thus not a coefficient of performance (COP). In a recent review, Moya *et al*. noted the lack of a COP in the field of MCE materials [23] and a year later they proposed the coefficient *η* [24]:
3.2
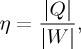
where *Q*=Δ*ST*_0_, with *T*_0_ the temperature at which Δ*S* was derived and *W* either the electrical work required to generate the field *H*_0_ in a solenoid or the mechanical work to move the sample into the field of a permanent magnet device. This coefficient is dimensionless; however, as we have shown in a recent study [25], in the case of first-order materials, even if one observes only limited hysteresis, it is not sufficient just to take Δ*S* and *T*_0_ as cooling metric. It turns out that the effect of hysteresis on the cyclic response of a material strongly depends on the shift of the critical temperature in response to an applied field. Materials with the same Δ*S* and hysteresis can show very different cyclic responses. Therefore, one has also to take the reversible adiabatic temperature change into account. Therefore, we propose to use CRP:
3.3
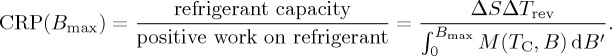
In their original proposal for CRP, Wood & Potter considered only second-order materials at cryogenic temperatures, and for these materials, Δ*S* and Δ*T* are fully reversible. They could therefore use mean-field theory to evaluate CRP and find values of about 2/3 in high fields *B*/*T*_0_≥1 and for lower fields CRP approaches zero [5]. The numerator in equation (3.3) is the cooling or net work of a reversible Carnot cycle, and therefore in a real machine with heat losses and the production of entropy, we shall always find lower performance. However, especially for first-order materials, the CRP helps in estimating how detrimental hysteresis effects are in a given material. Note that hysteresis effects were completely neglected in the Ashby maps proposed by Sandeman [26].

## 4. Application to room-temperature refrigerants

As mentioned above, Gd is considered as benchmark material for room-temperature magnetic refrigeration. Based on the data of Dan’kov *et al*. [6] for magnetization, Δ*S* and Δ*T*, we derive CRP values of 0.13, 0.17, 0.20 and 0.22 for field changes of 0.5, 1, 1.5 and 2 T, respectively. For values of the order of 2/3 as found by Wood & Potter in their calculations, one would need magnetic fields exceeding 10 T. Finding a complete set of data including magnetization and Δ*T* is rather important as the data should originate from the same sample or at least from the same batch. Otherwise, as was shown by Dan’kov *et al*. [6], even for second-order materials, small variations in impurity levels can have a drastic influence on *T*_C_ and the MCE. It is easiest to generate a complete set of data in-house, so we did so on samples of MnFe(P,X) with X=As, Ge and Si [25,27,28] and (Pr_0.65_Sr_0.35_)MnO_3_. Additionally, we looked at La(Fe,Si)_13_ [29], La(Fe,Mn,Si)_13_H_1.5_ [30] and at the barocaloric effect observed in FeRh [31]. The data are summarized in table 1, and for the calculation of the mechanical work in the barocaloric experiment, we used the X-ray density of Fe_0.49_Rh_0.51_ and the volume change of 1% given in [30].

**Table 1.** Δ*S*, Δ*T* and CRP of several materials as determined with equation (3.3) at 1 T field change; for FeRh the pressure change of 250 MPa was used in the barocaloric experiment. The error in CRP values for LaFeSi type materials is estimated to be somewhat larger as we had to digitize the magnetic measurements.

materials	Δ*S* (J kg^−1^ K^−1^)	Δ*T*_rev_ (K)	CRP	references
Mn_1.25_Fe_0.70_P_0.49_Si_0.51_	8.5	2.2	0.47(2)	this work
MnFe_0.95_P_0.58_B_0.078_Si_0.34_	13.0	3.1	0.78(2)	this work
Mn_1.20_Fe_0.80_P_0.75_Ge_0.25_	10.5	1.8	0.43(2)	this work
MnFeP_0.45_As_0.55_	8	2.9	0.64(2)	this work
MnFe_0.95_P_0.595_B_0.075_Si_0.33_	9.8	2.8	0.62(2)	[28]
Gd	3	2.5	0.17(2)	[5]
Pr_0.65_Sr_0.35_MnO_3_	2.4	1.15	0.10(1)	this work
LaFe_11.38_Si_1.26_Mn_0.36_H_1.52_	11.5	3.1	0.63(7)	[30]
LaFe_11.6_Si_1.4_	14	2	0.37(7)	[29]
Fe_0.49_Rh_0.51_ @ 250 MPa	11.2	5	0.22(2)	[31]

From table 1 we find that first-order MCE materials produce larger values of CRP than Gd. The only exception is the manganite, which shows a rather low Δ*T*. Here, the three oxygen atoms per formula unit contribute significantly to the specific heat that contributes inversely to Δ*T*. The systematically larger values of CRP for the MnFe(P,X) compounds with X=As and Si, B compared to the La(Fe,Si)_13_ compounds result from the completion of the metamagnetic transition, combined with a very low hysteresis for the former compounds. Seemingly, Mn substitution of Fe has some detrimental effect on the La(Fe,Si)_13_ hydride samples.

As the CRP requires a rather complete set of data, currently it is not possible for us to compare all caloric materials known in the literature. This paper is intended to encourage our peers to collect these complete sets and publish these data. As commercial refrigerators will operate in rather low fields, one should concentrate on data in magnetic fields around 1 T, pressures of a few hundred megapascals and electric fields below kilovolts.

## Data accessibility

The data provided in table 1 are part of the PhD thesis of H.Y., ‘Nature of the first-order magnetic phase transition in giant-magnetocaloric materials’, chapters 4 and 5. An electronic version of this dissertation is available at http://repository.tudelft.nl/.
